# dmLT Adjuvant Enhances Cytokine Responses to T Cell Stimuli, Whole Cell Vaccine Antigens and Lipopolysaccharide in Both Adults and Infants

**DOI:** 10.3389/fimmu.2021.654872

**Published:** 2021-05-14

**Authors:** Marjahan Akhtar, Nuder Nower Nizam, Salima Raiyan Basher, Lazina Hossain, Sarmin Akter, Taufiqur Rahman Bhuiyan, Firdausi Qadri, Anna Lundgren

**Affiliations:** ^1^ Infectious Diseases Division, icddr,b (International Centre for Diarrhoeal Disease Research, Bangladesh), Dhaka, Bangladesh; ^2^ Department of Microbiology and Immunology, Institute of Biomedicine, University of Gothenburg, Gothenburg, Sweden

**Keywords:** dmLT, adjuvant, T cell, monocyte, infants, lipopolysacharide, IL-17A, IL-1β

## Abstract

Enhancement of mucosal immune responses in children and infants using novel adjuvants such as double mutant heat labile toxin (dmLT) is an important goal in the enteric vaccine field. dmLT has been shown to enhance mucosal IgA responses to the oral inactivated enterotoxigenic *Escherichia coli* (ETEC) vaccine ETVAX. dmLT can enhance IL-17A production from adult T cells, which may increase the production and secretion of mucosal IgA antibodies. However, the adjuvant mechanism remains to be fully elucidated and might differ between infants and adults due to age-related differences in the development of the immune system. The main objective of this study was to determine how dmLT influences antigen presenting cells and T cells from infants compared to adults, and the role of IL-1β for mediating the adjuvant activity. Peripheral blood mononuclear cells (PBMCs) from Bangladeshi infants (6-11 months) and adults (18-40 years) were stimulated with the mitogen phytohaemagglutinin (PHA), the superantigen Staphylococcal enterotoxin B (SEB), ETVAX whole cell component (WCC) or *E. coli* lipopolysaccharide (LPS) ± dmLT, and cytokine production was measured using ELISA and electrochemiluminescence assays. The adjuvant dmLT significantly enhanced SEB- and PHA-induced IL-17A, but not IFN-γ responses, in PBMCs from both infants and adults. Blocking experiments using an IL-1 receptor antagonist demonstrated the importance of IL-1 signaling for the adjuvant effect. dmLT, ETVAX WCC and LPS induced dose-dependent IL-1β responses of comparable magnitudes in infant and adult cells. Depletion experiments suggested that IL-1β was mainly produced by monocytes. dmLT enhanced IL-1β responses to low doses of WCC and LPS, and the adjuvant effect appeared over a wider dose-range of WCC in infants. dmLT and WCC also induced IL-6, IL-23 and IL-12p70 production in both age groups and dmLT tended to particularly enhance IL-23 responses to WCC. Our results show that dmLT can induce IL-1β as well as other cytokines, which in turn may enhance IL-17A and potentially modulate other immunological responses in both infants and adults. Thus, dmLT may have an important function in promoting immune responses to the ETVAX vaccine, as well as other whole cell- or LPS-based vaccines in infants in low- and middle-income countries.

## Introduction

Enteric infections are important causes of morbidity and mortality, particularly in young children in low and middle-income countries, but so far only a limited number of licensed vaccines exist against such infections (1–3). Oral vaccines induce immune responses locally in the gastrointestinal mucosa, but the responses are often lower in young children and infants compared to in adults (1, 4, 5). Responses to oral vaccines are also generally lower in children in low-resource countries compared to in more developed parts of the world (4, 6). Therefore, enhancement of mucosal immune responses in children and infants using novel vaccine adjuvants that can be administered orally is an important goal in the field of enteric vaccination.

Enterotoxigenic *Escherichia coli* (ETEC) is one of the leading bacterial causes of watery diarrhea in children and infants in low- and middle-income countries. Several strategies are currently used to enhance the immunogenicity of ETEC vaccines, including the addition of the oral adjuvant double mutant heat-labile toxin (dmLT). Clements *et al.* developed dmLT from the heat labile toxin (LT) produced by ETEC and made it safe for use, without losing immunostimulatory properties, by altering two amino acids (R192G/L211A) in the enzymatically active A subunit of native LT (7–9). Oral administration of up to 250 µg of dmLT did not cause any enterotoxicity in a mouse assay, while ≥5 µg of native LT caused substantial intestinal fluid secretion (8). Importantly, dmLT has clear adjuvant properties, as demonstrated in several preclinical and clinical studies of mucosal vaccines, including ETEC, *Helicobacter pylori* and *Streptococcus pneumoniae* vaccines (7, 10–14). ETVAX is a leading oral ETEC vaccine candidate which consists of inactivated *E. coli* bacteria, overexpressing the four most prevalent ETEC colonization factors (CFs) CFA/I, CS3, CS5 and CS6, and a toxoid molecule (12, 13, 15). Recent results from clinical trials of ETVAX demonstrate promising adjuvant effects of dmLT on IgA responses in adults, as well as children of different ages (13, 16). dmLT has also been shown to improve the protection afforded by an oral live attenuated ETEC vaccine in adults in an experimental challenge study (14).

The adjuvant mechanism of dmLT is not entirely elucidated, but effects on antigen processing and presentation in antigen presenting cells (APCs) are likely to be of critical importance (7, 17). Moreover, dmLT triggers activation of caspase1/inflammasome signaling pathways and subsequently secretion of IL-1β, IL-23, IL-6 and other cytokines from APCs (7). These cytokines promote mixed Th1/Th2/Th17 responses in mice, with a particularly strong induction of Th17 cells (7). Importantly, Leach *et al.* and Larena *et al.* showed that dmLT, and the related multiple mutant cholera toxin (mmCT) adjuvant, can enhance human IL-17A cytokine responses in T helper cells from adults by activating cAMP-dependent protein kinase A and caspase1/inflammasome–dependent IL-1 signaling (18, 19). IL-17A is particularly important in antibody-mediated protection against enteric infections, enhancing germinal center formation, IgA and IgG class switching and secretory IgA formation by increasing the expression of the poly-Ig receptor in epithelial cells (20–24).

Although infants and children are the main target groups for most enteric vaccines, the adjuvant mechanism of dmLT remains unexplored in these age groups. Both innate and adaptive immune cells in infants are immature or may function differently compared to adult cells (25–27). For example, immune cells from infants have been reported to have a higher baseline production of cAMP, as well as higher production of IL-1β in response to some bacterial stimuli, compared to adult cells (28, 29), whereas production of several other cytokines (including IFN-γ and TNF-α) have consistently been reported to increase with age in infants and children (26). Ongoing efforts to evaluate the adjuvant function of dmLT in combination with different vaccines in different age groups, make it important to understand how the dmLT adjuvant may modulate immune responses in infants compared to adults.

In this study, we investigated if there are age-related differences in the capacity of the dmLT adjuvant to enhance cytokine responses in T cells and APCs, and analyzed the role of IL-1β for mediating the adjuvant function in infants compared to adults. We also analyzed if dmLT can modulate cytokine responses induced by the whole cell component (WCC) of ETVAX or *E. coli* lipopolysaccharide (LPS) in cells from adults and infants.

## Methods and Materials

### Study Participants

Healthy Bangladeshi adults (18-40 years; n=26) and infants (6-11 months; n=25) were enrolled from similar socio-economic conditions (**Table 1**). Informed written consent was obtained from each adult participant and from parent/guardian of the infants. Participants were enrolled based on the clinical assessment of study physicians. Key exclusion criteria were history of gastrointestinal disorders, diarrheal or febrile illness during the last two weeks and antibiotic treatment within one week prior to enrollment. The studies were approved by the research review and ethical review committees of the International Centre for Diarrhoeal Disease Research, Bangladesh (icddr,b).

**Table 1:** Demographic characteristics of study participants.

	Adults (n=26)	Infants (n=25)
**Age**		
Mean (SD)	26.5 (6.5)^a^	8.5 (1.8)^b^
Range	18-40^a^	6-11^b^
**Gender (no. and freq. of participants)**		
Male	7 (27%)	13 (52%)
Female	19 (73%)	12 (48%)

a Age in years.

b Age in months.

### Specimen Collection and Processing

Heparinized venous blood was collected on the day of enrollment. PBMCs were separated by density-gradient centrifugation using Ficoll-Isopaque (Pharmacia, Sweden). From subsets of participants, CD14+ monocytes were depleted from PBMCs using magnetic beads (Dynabeads, Dynal AS, Norway), according to the manufacturer´s instructions. The frequencies of CD14+ monocytes in PBMCs after depletion were <7% (mean 4%) in all experiments, as determined by flow cytometric analysis.

### Antigens, Polyclonal Stimuli and Adjuvant

PBMCs were stimulated with combinations of the following polyclonal stimuli, antigens, toxins or toxin derivatives: Staphylococcal enterotoxin B (SEB; Sigma-Aldrich, USA), phytohaemagglutinin (PHA, Remel, USA), *E. coli* O111:B4 LPS (Sigma Aldrich), dmLT (Scandinavian Biopharma, Sweden) and heat labile toxin B subunit (LTB, Scandinavian Biopharma). The cells were also stimulated with O78 LPS antigen, which was prepared in house from an enterotoxigenic O78 *E. coli* strain (H10407 P) by hot phenol water extraction followed by DNase and protease treatment, as described (30). The preparation contained less than 1% protein as determined by SDS page analyses using a standard protein as reference. Cells were also stimulated with a whole cell vaccine component (WCC, Scandinavian Biopharma, Etec970) corresponding to the composition of ETVAX, i.e. consisting of three formalin-inactivated O78 LPS-expressing LT and heat stabile toxin (ST) negative *E. coli* strains over-expressing CFA/I, CS3 and CS5 and one phenol inactivated *E. coli* K12 strain over-expressing CS6 (12, 13, 15, 16). The WCC did not contain any LCTB*A* or dmLT.

### Cell Stimulation

Cells were cultured in DMEM F12 medium (Thermo Fisher Scientific, USA), supplemented with 50 mg/mL gentamicin and 5% human AB^+^ serum, at 37°C in a 5% CO_2_ incubator. PBMCs and PBMCs depleted of CD14+ monocytes (10^5^ cells/well) were cultured in duplicate wells in U-bottomed 96-well plates. Cells were left untreated or stimulated with SEB (10 ng/ml) or PHA (1 µg/ml) alone or in combination with increasing concentrations (1 µg/ml or 10 µg/ml) of dmLT or LTB. After 72 h, supernatants were collected for cytokine analysis by ELISA (IL-17A and IFN-*γ*). For inhibition of IL-1 signaling, 1 µg/ml IL-1 receptor antagonist (IL-1RA, R&D Systems, USA) was added daily for three days to cell cultures. For determination of production of IL-1β, IL-6, IL-23, IL-12p70, IL-10 and TFN-α PBMCs or PBMCs depleted of CD14+ monocytes were left untreated or stimulated for 18 h with LPS from *E. coli* O111:B4, ETEC O78 LPS (both 0.1-1000 ng/ml) or ETVAX WCC (200-200,000 bacteria/ml) alone or in combination with dmLT (10 µg/ml), where after culture supernatants were collected. PBMCs were also stimulated with dmLT alone for 18 hours at increasing concentrations (1-50 µg/ml). All supernatants were stored at -70°C before cytokine analysis.

### Analysis of Cytokines

The concentrations of IL-1β (from 18 h culture supernatants), and IL-17A and IFN-γ (from 72 h culture supernatants), were determined using sandwich ELISA (eBioscience, USA), following the manufacturers’ instructions. The concentrations of IL-6, IL-23, IL-12p70, IL-10 and TFN-α (from 18 h culture supernatants) were determined using a multiplex electrochemiluminescence assay (V-plex, Meso Scale Discovery, USA).

### Statistical Analyses

Cytokine responses were evaluated using the Friedman test with Dunn’s multiple comparison post test, the Wilcoxon signed rank test or Mann-Whitney test, as applicable. *P*-values <0.05 were considered as significant. All statistical analyses were performed with GraphPad Prism (Graph Pad Software, USA) version 6.0.

## Results

### Effect of dmLT on IL-17A and IFN-γ Production

**Figure 1:**
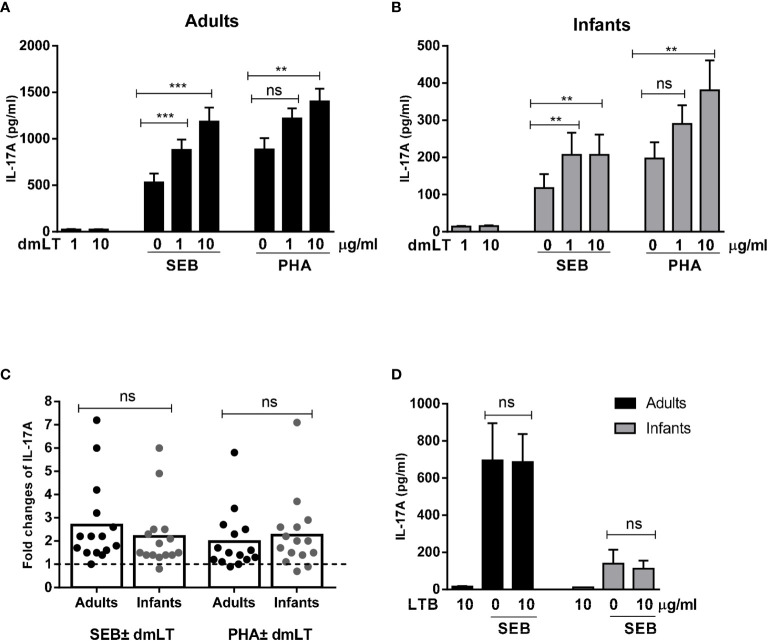
Effect of dmLT and LTB on IL-17A responses induced by SEB and PHA stimulation in PBMCs from adults and infants. IL-17A concentrations in cultures with cells from **(A)** adults (n = 15) and **(B)** infants (n = 15) stimulated with increasing concentrations of dmLT alone (1 or 10 µg/ml) or with SEB or PHA. **(C)** Fold changes in IL-17A concentrations in cultures with PBMCs from adults and infants stimulated with SEB or PHA plus dmLT (10 µg/ml) *versus* SEB or PHA stimulation alone. Bars represent means of fold-change differences and each symbol represents data from one participant. The dashed line indicates no enhancement (fold rise = 1). **(D)** Effect of LTB (10 µg/ml) on IL-17A responses induced by SEB in PBMCs from adults (n = 7) and infants (n = 7). **(A, B, D)** Bars represent mean with SEM of IL-17A concentrations in culture supernatants. Statistical analysis was performed using the Friedman test with Dunn’s multiple comparison post-test **(A, B)**, Mann-Whitney test **(C)** or Wilcoxon matched pairs sign rank test **(D)** ^**^ *P <* 0.01, ^***^ *P <* 0.001, ns, not significant; *P >* 0.05.

To study the influence of dmLT on T cell responses in cells from infants *versus* adults, PBMCs collected from the two different age groups were stimulated with dmLT in combination with the superantigen SEB or the mitogen PHA. Cells were stimulated polyclonally with SEB and PHA rather than with purified antigens to allow analysis of the adjuvant effect in all participants in a consistent manner, with limited influence of the infection or vaccination history of the individual. The adjuvant effect was evaluated by analyzing levels of IL-17A and IFN-γ in culture supernatants by ELISA after 72 h of stimulation. In cells from both adults and infants, SEB or PHA stimulation alone induced IL-17A responses, but the magnitudes of responses were lower in infants compared to adults (**Figures 1A, B**). In contrast, stimulation with dmLT alone did not induce any detectable IL-17A production at any concentration tested (1-10 µg/ml; **Figures 1A, B**).

Stimulation of cells with SEB in combination with increasing concentrations of dmLT, resulted in significantly enhanced IL-17A production compared to stimulation with SEB alone in cells from both adults and infants (**Figures 1A, B**). In adults, the production of IL-17A increased significantly in the presence of the lowest dmLT concentration tested (1 µg/ml, 2.1-fold mean increase compared to SEB alone) and addition of 10 µg/ml resulted in even higher IL-17A production (2.7-fold mean increase) (**Figure 1A**). PBMCs from adults also showed significantly increased IL-17A production when 10 µg/ml of dmLT was added in combination with PHA, and a trend for increased production was also observed at 1 µg/ml dmLT (**Figure 1A**).

In PBMCs from infants, significant IL-17A enhancing effects of dmLT were also observed when increasing concentrations of dmLT (1 and 10 µg/ml) were added to the cultures stimulated with SEB (**Figure 1B**). However, in contrast to adults, the two dmLT concentrations induced comparable IL-17A production. Similar to adults, dmLT 10 µg/ml caused significant IL-17A enhancement when combined with PHA stimulation in PBMCs from infants with a trend for increased responses seen already at 1 µg/ml (**Figure 1B**).

Since the levels of IL-17A production in response to PHA and SEB alone differed between cells from infants and adults, we also evaluated if the relative capacity of dmLT to enhance responses differed between infants and adults after normalization of the IL-17A levels induced by SEB or PHA stimulation alone. The relative increase (fold-rise) in IL-17A production in cells stimulated with 10 µg/ml dmLT compared to stimuli alone without dmLT were comparable in both age groups (**Figure 1C**, adults *vs.* infants mean fold rises: SEB 2.7 versus 2.2; PHA 2.0 versus 2.3), supporting that dmLT has a comparable adjuvant effect on T cell responses in the two age groups.

In contrast to the clear adjuvant effect observed when dmLT was added to the cultures, addition of 10 µg/ml of LTB, which spontaneously forms pentamers, but lack the enzymatically active A subunit, did not affect IL-17A responses to SEB in either adult or infant cells (**Figure 1D**). Responses to LTB alone were also very low.

**Figure 2:**
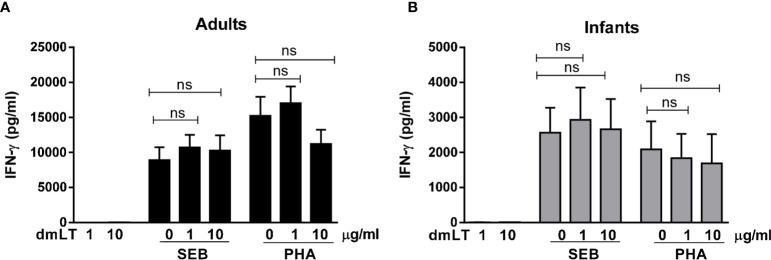
Effect of dmLT on IFN-γ responses induced by SEB and PHA stimulation in PBMCs from adults and infants. IFN-γ concentrations in cultures with cells from **(A)** adults (n = 15) and **(B)** infants (n = 15) stimulated with increasing concentrations of dmLT alone (1 or 10 µg/ml) or with SEB or PHA. Bars represent mean with SEM of IFN-γ concentrations in culture supernatants. Statistical analysis was performed using the Friedman test with Dunn’s multiple comparison post-test. ns, not significant; *P >* 0.05.

SEB and PHA also induced strong IFN-*γ* responses, with higher magnitudes of responses in PBMCs from adults compared to infants (**Figures 2A, B**). However, dmLT (**Figures 2A, B**) or LTB (**Supplementary Figure 1**) did not induce any IFN-*γ* production when added alone and did not enhance IFN-γ production in response to SEB or PHA in cells from either adults or infants.

Collectively, these results show that dmLT, in contrast to LTB, has a clear adjuvant effect on IL-17A responses in cells from both infants and adults, whereas no adjuvant effect was observed on IFN-*γ* responses in either of the age groups.

### Role of IL-1β in Promotion of IL-17A Responses

**Figure 3:**
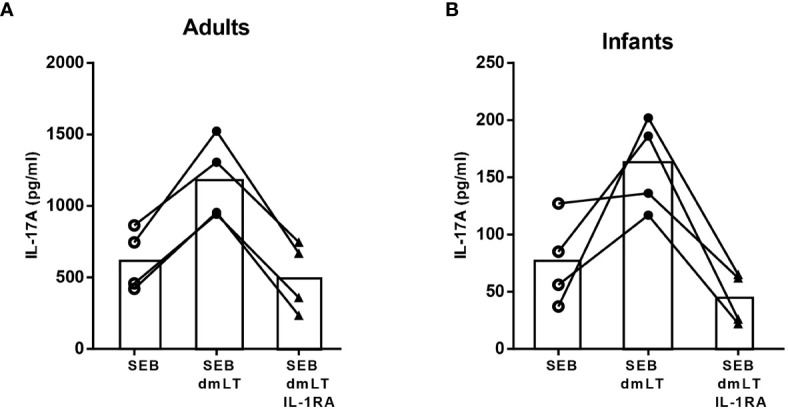
Role of IL-1 signaling in dmLT-induced promotion of IL-17A responses. IL-17A concentrations in cultures with PBMCs from **(A)** adults (n = 4) and **(B)** infants (n = 4) stimulated with SEB alone or SEB + 10 µg/mL dmLT with or without addition of IL-1 receptor antagonist (IL-1RA). Each symbol and line represents data from one participant.

To evaluate the role of IL-1β in mediating the dmLT adjuvant effect in adult and infant PBMCs, IL-1RA was added to cell cultures to prevent downstream effects of IL-1β by blocking the IL-1 receptor. Addition of IL-1RA to cells stimulated with SEB plus dmLT decreased the IL-17A production to levels corresponding to those measured in cultures stimulated with SEB alone in cells from all tested adults and infants (**Figure 3**). These results support an important role for IL-1β in the adjuvant effect of dmLT in both infants and adults.

### IL-1β Responses to dmLT, ETVAX WCC and LPS

**Figure 4:**
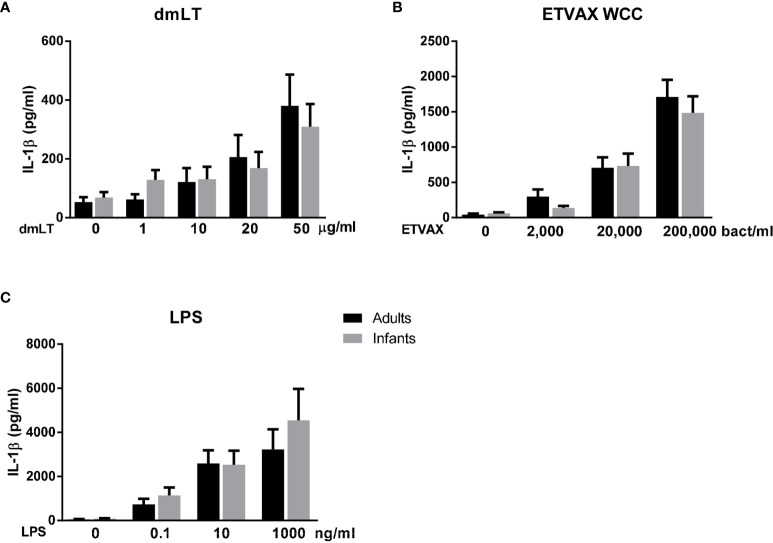
IL-1β production from PBMCs from adults and infants stimulated with dmLT or ETVAX WCC. IL-1β concentrations in cultures with cells from PBMCs from adults and infants with increasing concentrations of **(A)** dmLT (1-50 µg/ml, adults; n = 10, infants; n = 8), **(B)** ETVAX WCC (2000-200,000 bacteria/ml, adults; n=13, infants; n = 13) or **(C)** ETEC O78 LPS (0.1-1000 ng/ml, adults; n = 9, infants; n = 8). Bars represent means with SEM of concentrations of IL-1β in culture supernatants. *P >* 0.05 for comparisons of IL-1β concentrations in cultures with cells from adults *versus* infants at all stimuli concentrations tested (Mann-Whitney test).

To further investigate if dmLT alone could induce production of IL-1β in PBMCs from both adults and infants, PBMCs were stimulated with increasing concentrations of dmLT (1, 10, 20 and 50 µg/ml) for 18 hours, a time point when mainly innate cells respond to stimulation. dmLT alone activated PBMCs from both infants and adults to produce IL-1β in a dose-dependent manner (**Figure 4A**). The levels of IL-1β were comparable in adults and infants at all dmLT concentrations tested (*P*>0.05). Increased IL-1β production (≥2-fold increase compared to stimulation with medium alone) was already seen at the concentration of 10 µg/ml of dmLT in PBMCs from 56% of infants and 40% of adults. However, even stronger and more consistent IL-1β responses were observed at 50 µg/ml dmLT in cells from both adults (90%, 6.8 mean fold-rise) and infants (67%, 4.1 mean fold-rise, *P*>0.05, adults *versus* infants). Control experiments showed that stimulation with LTB induced little IL-1β production (data not shown).

Since dmLT has been shown to enhance immune responses to the inactivated ETEC vaccine ETVAX in several studies (12, 13, 16), we also investigated the role of IL-1β in responses to the bacterial component of ETVAX (whole cell component; WCC), which did not contain any LCTB*A* or dmLT. In both adult and infant cell cultures, stimulation with ETVAX WCC induced strong and dose-dependent IL-1β responses with comparable levels of IL-1β in the two age groups at all concentrations tested (*P*>0.05, **Figure 4B**). The IL-1β responses may at least partly be a result of stimulation by the LPS component of the WCC. To investigate if purified LPS also induced IL-β production, cells were stimulated with increasing concentrations of purified ETEC O78 LPS (0.1-100 ng/ml), expressed by three of the four *E. coli* strains in the ETVAX WCC and purified in-house. Strong dose-dependent IL-1β production was observed with similar levels produced in cells from infants and adults (**Figure 4C**). Comparable IL-1β responses were also observed in response to stimulation with commercially available *E. coli* O111:B4 LPS in both age groups (**Supplementary Figure 2**).

**Figure 5:**
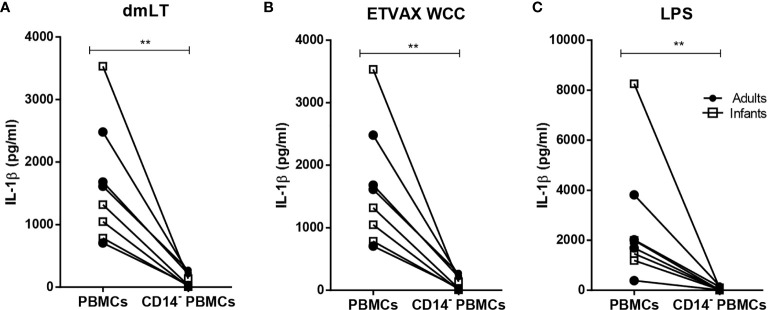
IL-1β production from PBMCs depleted of CD14+ monocytes in response to dmLT, ETVAX WCC and LPS. IL-1β concentrations in cultures with PBMCs and PBMCs depleted of CD14^+^ monocytes from adults (n = 4, closed symbols) and infants (n = 5, open symbols) stimulated with **(A)** dmLT (20 µg/ml), **(B)** ETVAX WCC (200,000 bacteria/ml) or **(C)** *E*. *coli.* O111:B4 LPS (10 ng/ml). Each symbol and line represent data from one participant. Statistical analysis was performed using the Wilcoxon matched-pairs signed rank test. ^**^ *P <* 0.01.

Depletion of CD14^+^ monocytes from PBMCs isolated from both adults and infants resulted in almost complete loss of IL-1β production in response to stimulation with dmLT, ETVAX WCC or *E. coli* LPS (**Figure 5**).

Collectively, these results demonstrate that dmLT alone, as well as ETVAX WCC and *E. coli* LPS, induce dose-dependent IL-1β responses of comparable magnitudes in PBMCs from infants and adults, and suggest that IL-1β was mainly produced by monocytes in both age groups.

### IL-1β Production in PBMCs Stimulated With ETVAX WCC or LPS ± dmLT

**Figure 6:**
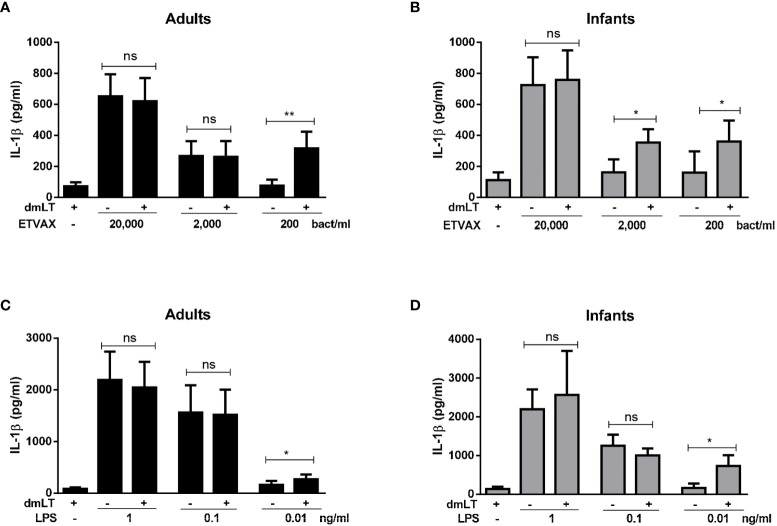
IL-1β production from PBMCs stimulated with ETVAX WCC or LPS ± dmLT. IL-1 β concentrations in cultures with PBMCs from **(A, C)** adults and **(B, D)** infants stimulated with increasing concentrations of ETVAX WCC (200-20,000 bacteria/ml) or ETEC O78 LPS (0.01-1 ng/ml) alone or with 10 µg/ml dmLT. Bars represent mean with SEM of IL-1β concentrations in culture supernatants (ETEC WCC 2000 bacteria/ml; adults n = 13, infants n = 14, 200 bacteria/ml; adults n = 9, infants n = 9, LPS 0.1 ng/ml; adults n = 12, infants n = 11, LPS 0.01 ng/ml; adults n=6, infants n=6). Statistical analysis was performed with the Wilcoxon matched-pairs signed rank test. ^*^ *P <* 0.05, ^**^ *P <* 0.01, ns, not significant; *P >* 0.05.

To determine if dmLT can further enhance IL-1β responses induced by ETVAX WCC in PBMCs from adults and infants, IL-1β responses were measured after addition of 10 µg/ml of dmLT with different doses of ETVAX WCC (200-200 000 bacteria/ml). In cells from adults, dmLT only enhanced responses to the lowest WCC concentration tested (200 bacteria/ml, **Figure 6A**). In contrast, in infant cells dmLT enhanced IL-1β responses to both 200 and 2000 bacteria/ml (**Figure 6B**). dmLT did not influence IL-1β responses to higher doses of ETVAX WCC (20 000 bacteria/ml, **Figures 6A, B**, or 200 000 bacteria/ml, data not shown).

Next, we tested whether dmLT may also influence IL-1β responses to purified LPS. PBMCs were stimulated with increasing concentrations of ETEC O78 LPS (0.01-1 ng/ml) alone and in combination with 10 µg/ml of dmLT. In cells from both adults and infants, significantly enhanced IL-1β responses were observed only when dmLT was added with the lowest tested concentration of O78 LPS (0.01ng/ml) compared to LPS alone (**Figures 6C, D**).

These results suggest that dmLT can enhance IL-1β responses induced by very low doses of inactivated *E. coli* bacteria, as shown by responses to ETVAX WCC, as well as to low concentrations of purified *E. coli* LPS. Our results also indicate that the adjuvant effect may be apparent over a wider dose-range of bacteria in PBMCs from infants compared to adults.

### Expanded Analysis of Cytokines Induced by dmLT and ETVAX WCC

To determine if dmLT and ETVAX induces additional early cytokine responses, which may modulate Th17 and Th1 and other adaptive immune responses, concentrations of IL-6, IL-23, IL-12p70, IL-10 and TNF-α were analyzed in a subset of samples collected after 18 h of stimulation. For this multiplex analysis, only individuals with a clear dose-dependent increase in IL-1β production in response to dmLT and ETVAX were included (adults n=5, infants n=5).

**Figure 7:**
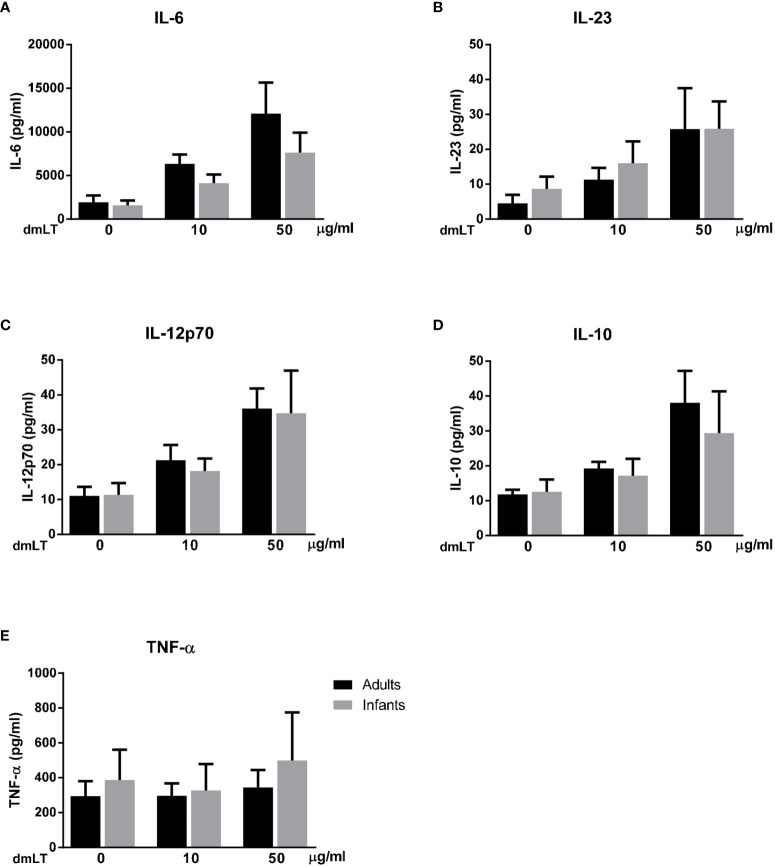
Production of IL-6, IL-23, IL-12p70, IL-10 and TNF-α from PBMCs stimulated with dmLT. Cytokine concentrations in cultures with PBMCs from adults (n = 5) and infants (n = 5) stimulated with increasing concentrations of dmLT (10 and 50 µg/ml). Bars represent mean with SEM of concentrations of **(A)** IL-6, **(B)** IL-23, **(C)** IL-12p70, **(D)** IL-10 and **(E)** TNF-α in culture supernatants.

We found that dmLT induced high production of IL-6 and lower production of IL-23, IL-12p70 and IL-10 (**Figures 7A–D**) in parallel to the IL-1β responses, in cells from both adults and infants (IL-1β responses in the selected sample subset are shown in **Supplementary Figure 3A**). A majority of adults and infants responded with increased production of IL-6, IL-23, IL-12p70 and IL-10 when stimulated with 10 µg/ml dmLT compared to levels detected in unstimulated cultures, with even stronger responses observed at 50 µg/ml in most participants. dmLT stimulation did not induce any consistent TNF-α responses in either infant or adult cells (**Figure 7E**).

**Figure 8:**
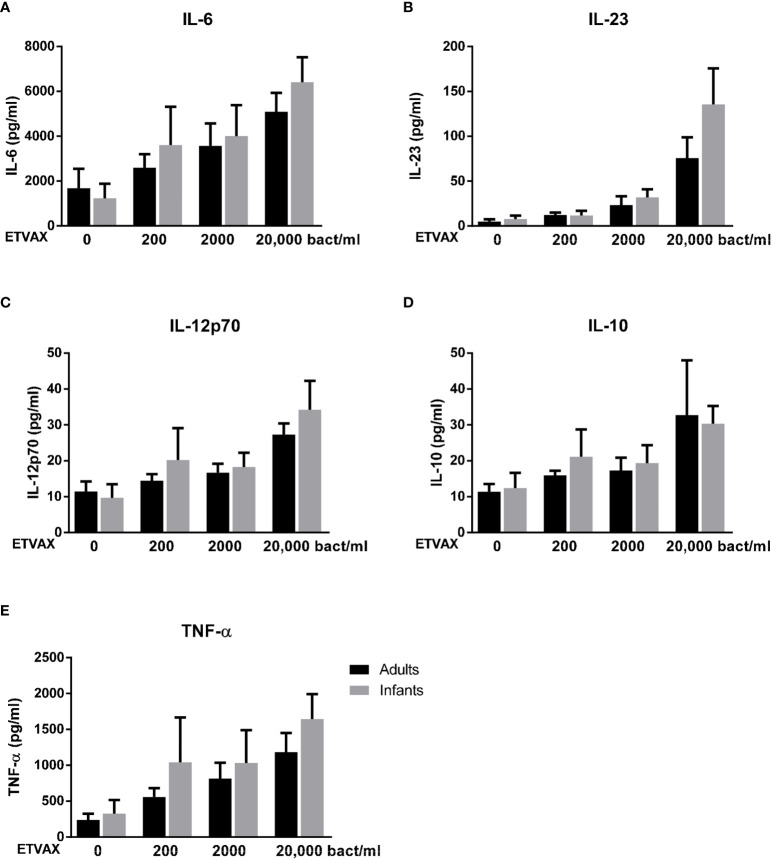
Production of IL-6, IL-23, IL-12p70, IL-10 and TNF-α from PBMCs stimulated with ETVAX WCC. Cytokine concentrations in cultures with PBMCs from adults (n = 5) and infants (n = 5) stimulated with increasing concentrations of ETVAX WCC (200-20 000 bacteria/ml). Bars represent mean with SEM of concentrations of **(A)** IL-6, **(B)** IL-23, **(C)** IL-12p70, **(D)** IL-10 and **(E)** TNF-α in culture supernatants.

Stimulation with ETVAX WCC induced strong, dose-dependent IL-6 and TNF-α responses in both age groups (**Figures 8A, E**). IL-23, IL-12p70 and IL-10 responses were lower and mainly detected at higher concentrations of bacterial stimuli (**Figures 8B–D**).

No clear differences in cytokine responses between cells from adults and infants were observed, although infant cells tended to produce more IL-23 in response to the highest tested concentration of ETVAX WCC (**Figure 8B**).

**Figure 9:**
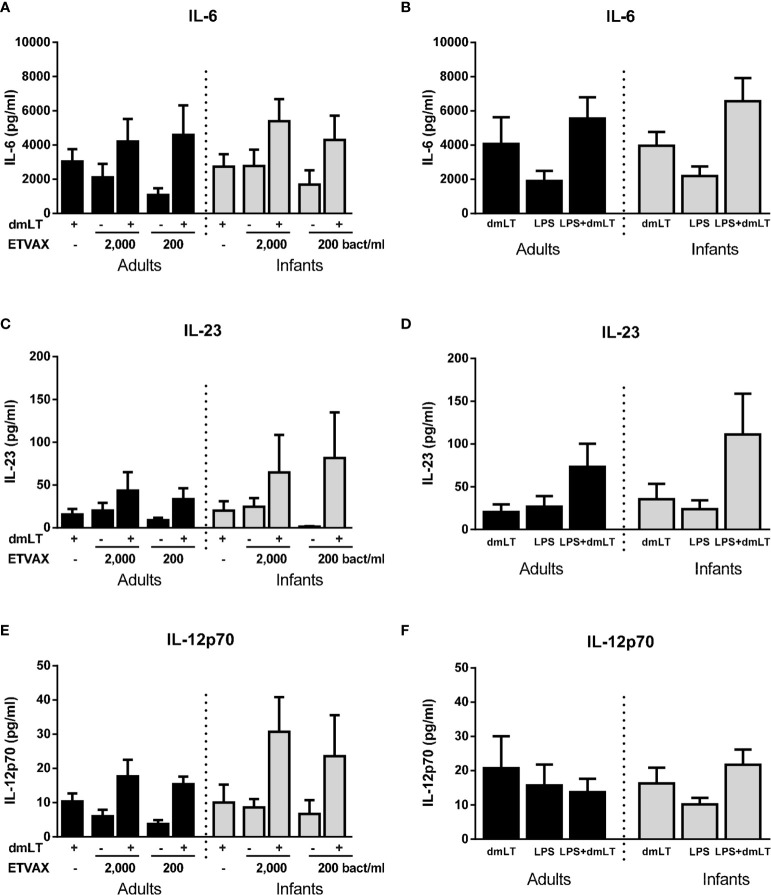
Production of IL-6, IL-23 and IL-12p70 from PBMCs stimulated with ETVAX WCC ± dmLT. **(A, B)** IL-6, **(C, D)** IL-23 and **(E, F)** IL-12p70 concentrations in cultures with PBMCs from adults (n = 5) and infants (n = 5) stimulated with ETVAX WCC (200 or 2000 bacteria/ml) or ETEC O78LPS (0.01 ng/ml) alone or with 10 µg/ml dmLT. Bars represent mean with SEM of cytokine concentrations in culture supernatants.

Finally, we investigated if the addition of dmLT could provide a broad enhancement of cytokine responses induced by ETVAX WCC or O78 LPS. In this subset analysis, only subjects who responded with ≥2-fold increase in IL-1β production to 10 µg/ml dmLT compared to IL-1β levels produced by unstimulated cells were included (adults n=5, infants n=5). IL-6 responses to low concentrations of both ETVAX WCC and LPS were increased by the addition of dmLT, but only to the extent expected by adding the responses to dmLT and ETVAX/LPS together in most subjects (**Figures 9A, B**). In contrast, dmLT appeared to enhance IL-23 responses to both ETVAX WCC and LPS beyond that expected by of a mere additive effect of combining the two stimuli (**Figures 9C, D**). This is similar to the enhancing effect observed for IL-1β (**Figure 6** and **Supplementary Figures 3C, D**). The trend for enhanced IL-23 responses with dmLT was particularly pronounced in infant cells. dmLT also tended to enhance IL-12p70 responses to ETVAX WCC in infants, but not in adults, and no enhancement was observed for LPS responses (**Figures 9E, F**). Consistent enhancement beyond an additive effect was not seen for IL-10 or TNF-α for either ETVAX WCC or LPS (**Supplementary Figures 4A, B**). dmLT tended to decrease TNF-α responses to both ETVAX WCC and LPS (**Supplementary Figures 4C, D**).

Results from control experiments, where responses in PBMCs depleted of CD14+ monocytes were compared with responses of the original PBMC population, suggested that the IL-6, IL-23, IL-12p70, IL-10 and TNF-α measured after stimulation with dmLT, ETVAX WCC or LPS were derived from monocytes (**Supplementary Figure 5**).

The results indicate that dmLT and ETVAX WCC alone and together can induce production of a range of cytokines from monocytes, which may have broad modulatory effects on adaptive immune responses in both adults and infants.

## Discussion

The mucosal adjuvant dmLT is one of the most well studied non-toxic substances safe for oral administration and can induce and promote multidimensional immune responses to co-delivered antigens (7). Most studies of dmLT and related adjuvants have been performed in mice *in vivo*, or *in vitro* using cell lines or cultured mouse cells, and only few studies have been done using human cells (7). In this study, we showed for the first time that PBMCs from infants and adults had similar capacity to be modulated by dmLT *in vitro*. dmLT promoted IL-17A production from PBMCs isolated from both infants and adults stimulated with SEB or PHA. In addition, our results showed that dmLT induced strong and comparable dose-dependent IL-1β responses in cells from adults and infants, and that the IL-17A promoting effect was at least partially mediated by IL-1β in both age-groups.

Although dmLT enhanced IL-17A responses in PBMCs from both adults and infants, responses to PHA and SEB were of lower magnitude in cells obtained from infants compared to adults. Previous studies using cells from Swedish adults clearly demonstrated that IL-17A was primarily produced by CD4+ T cells after stimulation with PHA and SEB with or without dmLT (18, 19). Since IL-17A is produced mainly by memory T cells (31, 32), the lower IL-17A production in infants is likely partly due to lower frequencies of memory CD4+ T cells present in infant compared to adult PBMCs, as previously demonstrated in participants from the same study area (29). The dose-response effect of dmLT was also more pronounced in response to PHA than SEB in infant cells. Previous studies have shown age-related differences in how CD4+ T cells respond to SEB (33, 34), whereas infant T cells responded to PHA with comparable proliferation compared to adult cells (35), suggesting that PHA may provide a more robust *in vitro* stimulation system when comparing responses in different age groups. Importantly, when the relative increase (fold-rises) in IL-17A production were analyzed in cells stimulated with PHA or SEB plus dmLT compared to polyclonal stimulation alone, a comparable relative enhancement of IL-17A responses was observed in both infants and adults and for both types of stimuli. These results suggest that cells from infants have a similar functional capacity as adult cells to be stimulated by dmLT.

Enhanced IL-17A responses may have important downstream consequences on mucosal immune responses. One important function of IL-17A appears to be the clearance of extracellular pathogens during infections by promoting the upregulation of poly Ig receptor on the epithelial cells, thus increasing IgA secretion in the lumen (20). IL-17A is also required for IgA and IgG antibody production, as demonstrated by a significant impairment of mucosal IgA and systemic IgA and IgG1 production after oral immunization of IL-17A deficient mice (36). Moreover, IL-17A can promote fibroblasts and epithelial cells to secrete pro-inflammatory mediators, including G-CSF, GM-CSF, and IL-8, which increase the production and release of neutrophils from the bone marrow and tissue recruitment (37, 38). IL-17A can also induce monocyte migration to tissues both directly and *via* induction of chemokines such as CCL2 (39). By promoting IL-17A production, dmLT may thus enhance both innate immunity and humoral responses, which may increase the protection against mucosal infections.

We also showed that LTB, in contrast to dmLT, did not affect IL-17A responses in cells from either infants or adults. This is consistent with our previous demonstration that both CTB and LTB lack adjuvant activity in cells from adult Swedes (18, 19) and with previous *in vitro* and *in vivo* studies showing reduced or completely blocked immunological effects using LTB or LT variants with mutations in critical positions in the A subunit (7).

Our results showed considerably lower IL-17A responses after the addition of IL-1 receptor antagonist in PBMC cultures stimulated with SEB plus dmLT, demonstrating the importance of IL-1β in mediating dmLT adjuvant function. The role of IL-1β was also evident from earlier experiments showing that inhibition of the molecules involved in IL-1β production from APCs, e.g. protein kinase A and caspase 1, caused significantly diminished adjuvant effect of dmLT (19). Several studies have demonstrated the potential role of IL-1β, in combination with IL-6 and/or IL-23, for the differentiation of human Th17 cells from naïve T cells, as well as promotion of IL-17A production from already differentiated Th17 memory T cells (40, 41). APCs, mostly monocytes and DCs, play major role in Th17 differentiation by producing IL-1β, IL-23 and IL-6 (40, 42, 43). In our study, we also showed that PBMCs from infants produced IL-1β at levels similar to adults at all concentrations of dmLT tested (1-50 µg/ml). We further showed that stimulation with ETVAX or LPS alone induced strong dose-dependent IL-1β production of comparable magnitudes in PBMCs from both adults and children. Depletion experiments verified that most of the IL-1β detected in the supernatants after stimulation with dmLT, ETVAX or LPS was derived from monocytes. Our previous studies have demonstrated similar proportions of CD14+ monocytes in PBMCs isolated from infants and adults from the same study area (29), suggesting that monocytes from infants and adults have comparable ability to respond to the stimuli tested. 

A key finding in this study was the ability of dmLT to enhance IL-1β responses to ETVAX whole cell component and purified *E. coli* LPS in both age groups. However, the enhancement was only observed at very low stimuli concentrations. Thus, it is clear that LPS, and potentially other bacterial components of the vaccine, have a potent capacity to induce IL-1β production on their own, but when the stimuli concentrations were reduced, dmLT could achieve an adjuvant effect. The adjuvant effect was also apparent over a wider dose-range of bacteria when stimulating cells from infants compared to from adults. Although the adjuvant effect was only observed at very low concentrations of bacteria and LPS, which are difficult to directly translate into *in vivo* conditions, this is an interesting observation, particularly since fractionated doses of ETVAX are given to children in order to avoid side effects such as vomiting. The amount of vaccine reaching individual immune cells in the mucosa is likely to be low, supporting the relevance of our *in vitro* findings. Consistent with these *in vitro* results, co-administration of dmLT with fractionated ETVAX doses was recently shown to significantly enhance IgA responses to O78 LPS in Bangladeshi infants (Svennerholm, A-M., unpublished results).

Although our study focused on IL-17A responses and the role of IL-1β for induction of such responses, we also analyzed whether dmLT and ETVAX WCC induced a broader range of cytokines related to Th17, Th1, immunosuppression and inflammation in a subset of samples. We found that dmLT and ETVAX WCC induced production of relatively high concentrations of IL-6 and lower but consistent production of IL-23. The adjuvant effect of dmLT when added together with ETVAX WCC was particularly pronounced for IL-23 responses in infants. This is interesting, considering the involvement of IL-23 in the induction and/or maintenance of Th17 cells (40, 41). We have previously shown that neutralization of IL-23, but not IL-6, can partially block the adjuvant effect of dmLT on IL-17A responses in cells from adults *in vitro*, although the blocking effect of IL-1 neutralization was more complete (18). The relative importance of IL-1β and IL-23 in the adjuvant function of dmLT should be investigated in further detail in continued studies.

We observed induction of IL-12p70 in response to both dmLT and ETVAX WCC, and a trend for enhanced responses when dmLT was combined with ETVAX WCC in cultures with infant cells. IL-12 is important for induction of Th1 responses and our results support that these responses may also be influenced by dmLT, particularly in infants. However, in contrast to the clear adjuvant effect of dmLT on IL-17A responses to polyclonal T cell stimuli, dmLT had no effect on IFN-*γ* responses, neither in cells from infants nor adults, in our study. These results are consistent with previous findings using cells from Swedish adults, where dmLT strongly supported IL-17A, but not IFN-*γ* responses elicited by stimulation with PHA, SEB, pneumococcal whole cell antigen or mycobacterial purified protein derivative (18, 19). However, dmLT has been shown to promote both IL-17A and IFN-γ production in response to LTB stimulation of cells derived from Swedish adults immunized with a prototype ETEC vaccine (18). Studies have shown that dmLT can enhance Th1 *in vivo* responses to some vaccines in mice, although the effect on Th17 responses have been more consistently observed across different studies (7). Thus, the ability of dmLT to affect Th1 responses may vary depending on antigen type, potentially having a greater ability to modulate responses to purified protein antigens such as LTB or tetanus toxoid, than to more complex antigen preparations, which may contain microbial components with intrinsic immunomodulatory properties. Further studies are needed to evaluate the *in vitro* adjuvant effect of dmLT on Th1 and Th17 responses to purified ETEC antigens, such as colonization factors, in infants. However, due to the limited blood volumes available from infants, and the transient nature of T cell responses in peripheral blood after mucosal infection or vaccination, such studies are challenging to perform and we did not have access to samples that allowed such analysis in this study.

dmLT and ETVAX WCC at high concentrations also induced IL-10, which has mainly immunosuppressive functions (44). In accordance with previous observations from studies of cholera toxin and LT (45, 46), dmLT appeared to suppress rather than enhance TNF-α responses to LPS or ETVAX WCC. Our results thus suggest that dmLT may induce a broad range of cytokines in addition to IL-1β from innate cells, but may also suppress the production of other cytokines. It is also clear that ETVAX WCC and *E. coli* LPS induced broad cytokine responses even in the absence of the adjuvant, and that the effect of dmLT is likely to be strongest at low antigen concentrations. However, as we only analyzed a limited number of cytokines in a relatively small number of samples, additional studies are needed to fully characterize the breadth of cytokine responses induced and modulated by dmLT and ETVAX. Our recent clinical demonstration that dmLT is safe in infants and children, and that dmLT can enhance IgA responses to LPS, are supporting the potential of this adjuvant for infant vaccination (16, and Svennerholm A-M et al, unpublished results). It will be important to further study the dose-response relationship of dmLT in combination with different vaccines both *in vitro* and *in vivo* and in different age groups to identify optimal combinations of adjuvant and antigen doses, which induces sufficient proinflammatory cytokines to drive protective responses, but do not cause any adverse or suppressive effects.

Collectively, our results suggest that dmLT can promote both innate and adaptive immune responses in infants. Enhanced production of IL-1β and other cytokines primarily produced by innate cells such as IL-23, may lead to increased IL-17A responses with subsequently enhanced production and secretion of secretory IgA. These responses may help to protect against ETEC and other mucosal infections. The ability of dmLT to enhance cytokine responses to low doses of ETVAX WCC and LPS in infants suggests that dmLT may also be used to overcome hyporesponsiveness to other whole cell- or LPS-based vaccines such as cholera, *Salmonella Typhi* and *Shigella* vaccines in infants in low- and middle-income countries. This will be an important target for investigation in future *in vitro* as well as *in vivo* studies. Our results thus encourage further evaluation of dmLT as an adjuvant to promote vaccine induced responses in infants.

## Data Availability Statement

The raw data supporting the conclusions of this article will be made available by the authors, without undue reservation.

## Ethics Statement

The studies involving human participants were reviewed and approved by Ethical review committee of the International Centre for Diarrhoeal Disease Research, Bangladesh. Written informed consent to participate in this study was provided by the participants’ legal guardian/next of kin.

## Author Contributions

AL, MA, TB, and FQ designed and planned the studies. MA, NN, SB, LH, and SA performed the immunological analyses. MA and AL wrote the manuscript. All authors contributed to the article and approved the submitted version.

## Funding

This work was supported by the European Union Seventh Framework Programme (FP7/2007–2013, grant agreement no. 261472 STOPENTERICS), the Swedish Research Council (grant number VR 348-2014-4228) and PATH through its enteric vaccine project. The funders had no role in study design, data collection or analysis, decision to publish, or preparation of the manuscript. icddr,b is thankful to the donors for their support to its research efforts. icddr,b also gratefully acknowledges the following donors who provided unrestricted support: Governments of Bangladesh, Canada, Sweden and the UK.

## Conflict of Interest

The authors declare that the research was conducted in the absence of any commercial or financial relationships that could be construed as a potential conflict of interest.

## Abbreviations

APC, antigen presenting cell; dmLT, double mutant heat labile toxin; ETEC, enterotoxigenic *Escherichia coli*; IL-1RA, IL-1 receptor antagonist; LPS, lipopolysaccharide; LT, heat labile toxin; LTB, heat labile toxin B subunit; PBMCs, peripheral blood mononuclear cells; PHA, phytohaemagglutinin; SEB, Staphylococcal enterotoxin B; WCC, whole cell component.
