# Supplementary material for: dmLT Adjuvant Enhances Cytokine Responses to T Cell Stimuli, Whole Cell Vaccine Antigens and Lipopolysaccharide in Both Adults and Infants
Source: Front Immunol. 2021 May 14;12:654872. doi: 10.3389/fimmu.2021.654872 (PMC8160295; doi:10.3389/fimmu.2021.654872)
Supplement: Supplementary file 1 [file DataSheet_1.pdf]

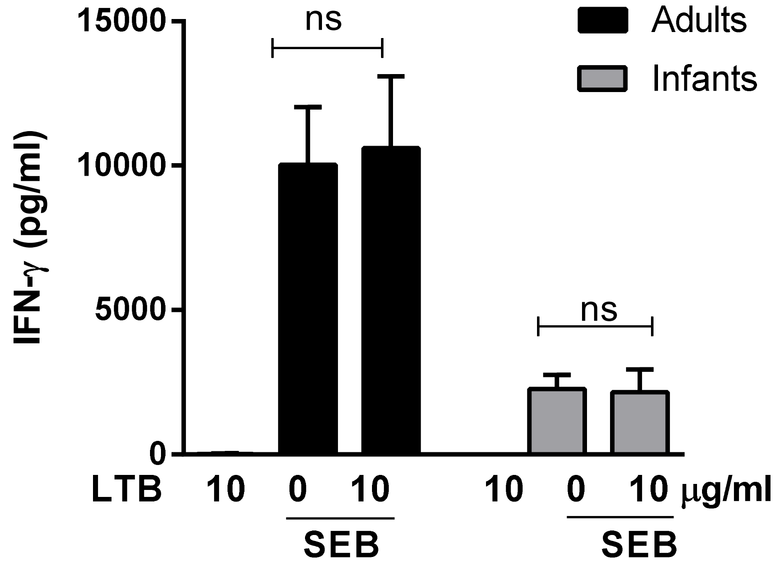


**Supplementary Figure 1. Effect of LTB on IFN-γ responses induced by SEB stimulation in PBMCs from adults and infants.** IFN-γ concentrations in cultures with cells from (**A**) adults (n=7) and (**B**) infants (n=7) stimulated with LTB (10 μg/ml) alone or with SEB. Bars represent mean with SEM of IFN-γ concentrations in culture supernatants. Statistical analysis was performed using Wilcoxon matched pairs sign rank test. ns; not significant; *P* >0.05.

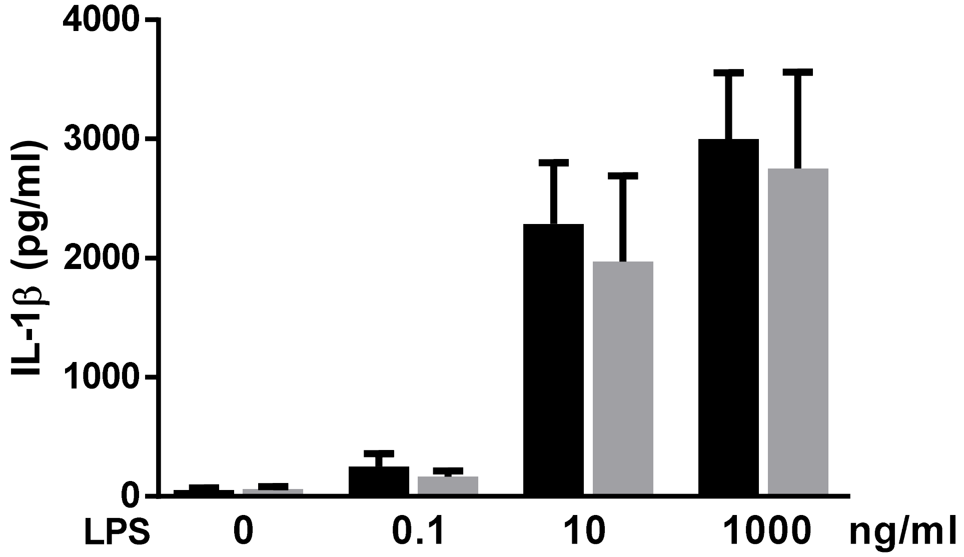


**Supplementary Figure 2. IL-1β production from PBMCs from adults and infants stimulated with commercially available *E. coli* O111:B4 LPS.** IL-1β concentrations in cultures with cells from PBMCs from adults and infants with increasing concentrations of *E. coli* O111:B4 LPS (0.1-1000 ng/ml, adults; n=9, infants; n=10). Bars represent means with SEM of concentrations of IL-1β in culture supernatants. *P*>0.05 for comparisons of IL-1β concentrations in cultures with cells from adults versus infants at all stimuli concentrations tested (Mann-Whitney test).

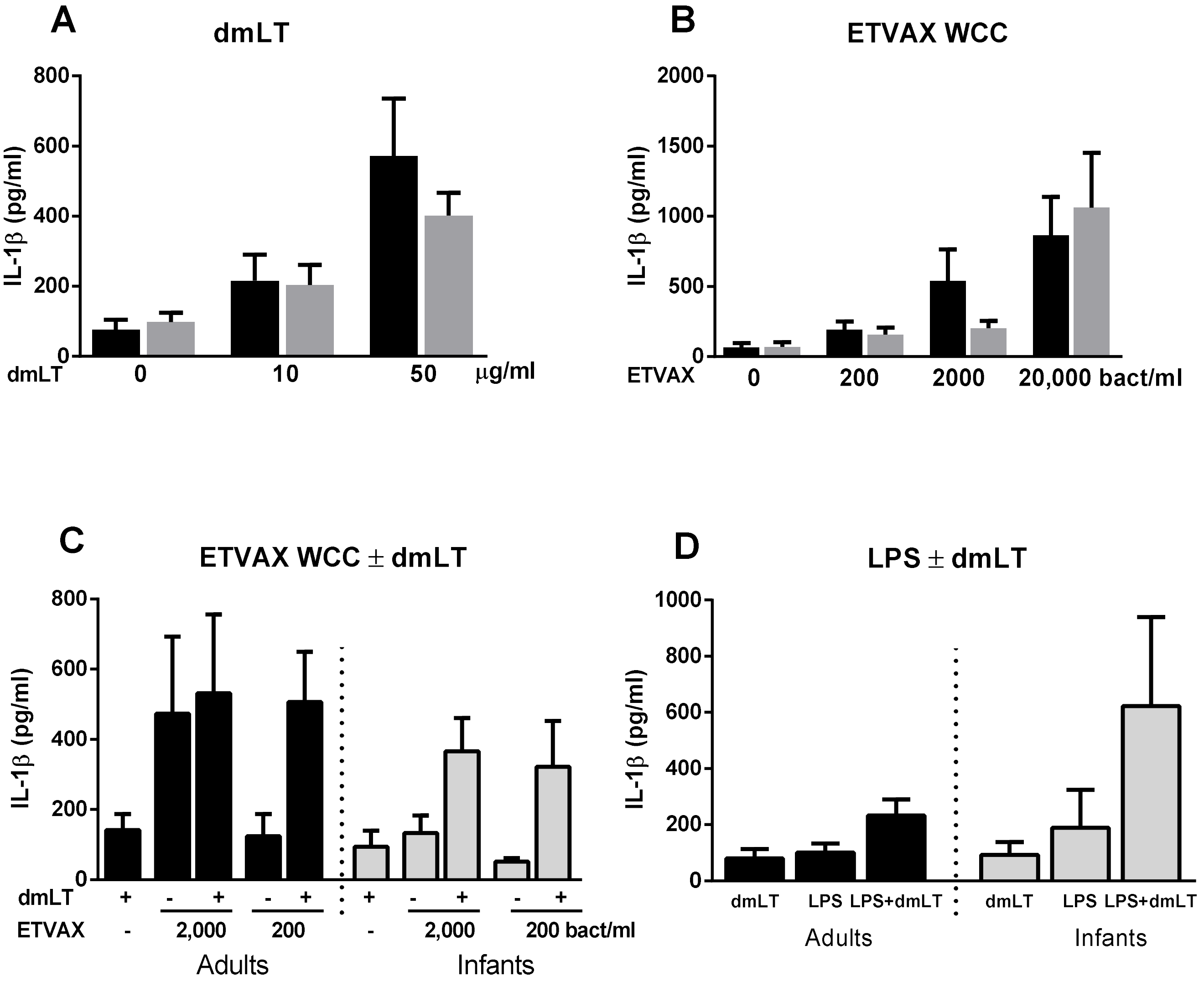


**Supplementary figure 3. IL-1β production from PBMCs from adults and infants stimulated stimulated with ETVAX WCC or O78 LPS ± dmLT.** IL-1β concentrations in cultures with cells from PBMCs from a subset of adults (n=5) and infants (n=5) stimulated with (**A**) dmLT (10 and 50 μg/ml), (**B**) ETVAX WCC (200-200,000 bacteria/ml), (**C**) ETVAX WCC with or without dmLT (10 μg/ml) or (**D**) O78 LPS (0.01 ng/ml) with or without dmLT (10 μg/ml). Bars represent means with SEM of concentrations of IL-1β in culture supernatants.

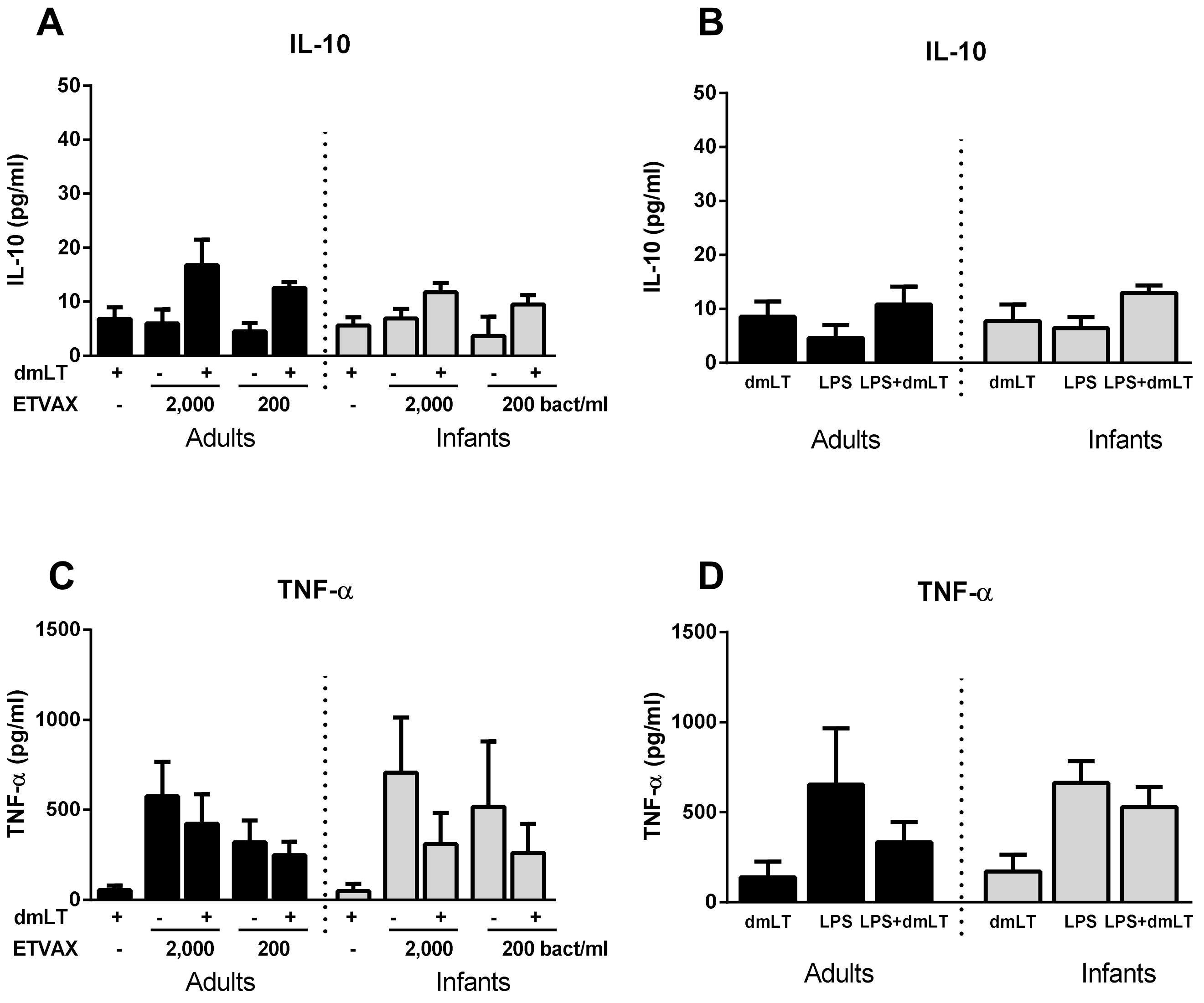


**Supplementary figure 4. Production of IL-10 and TNF-α from PBMCs from adults and infants stimulated with ETVAX WCC or O78 LPS ± dmLT.** (**A** and **B**) IL-10 and (**C** and **D**) TNF-α concentrations in cultures with PBMCs from adults (n=5) and infants (n=5) stimulated with (**A** and **C**) ETVAX WCC (200 or 2000 bacteria/ml) or (**B** and **D**) ETEC O78 LPS (0.01 ng/ml) alone or with 10 μg/ml dmLT. Bars represent mean with SEM of cytokine concentrations in culture supernatants.

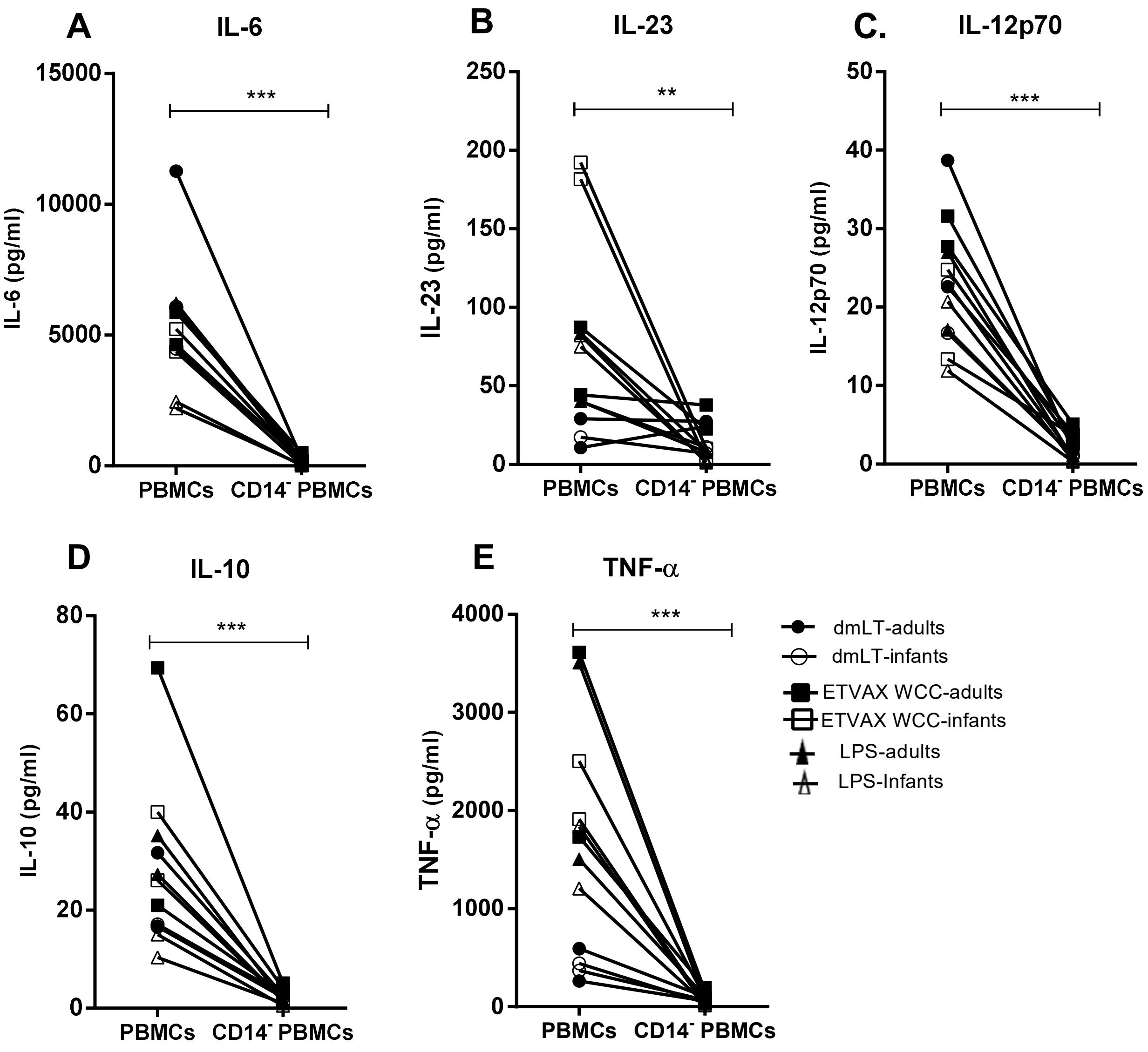


**Supplementary figure 5. Production of IL-6, IL-23, IL-12p70, IL-10 and TNF-α from PBMCs depleted of CD14+ monocytes in response to dmLT, ETVAX WCC and LPS.** Cytokine concentrations in cultures with PBMCs and PBMCs depleted of $CD14^{+}$ monocytes from adults (n=2, closed symbols) and infants (n=2, open symbols) stimulated with dmLT (20 µg/ml, circles), ETVAX WCC (200,000 bacteria/ml, squares) or *E. coli.* O111:B4 LPS (10 ng/ml, triangles). Each symbol and line represents data from one participant. Statistical analysis was performed using the Wilcoxon matched-pairs signed rank test. $^{**}P<0.01$, $^{***}P<0.001$.
